# Pathobiology of Anaplastic Large Cell Lymphoma

**DOI:** 10.1155/2010/345053

**Published:** 2011-02-06

**Authors:** Pier Paolo Piccaluga, Anna Gazzola, Claudia Mannu, Claudio Agostinelli, Francesco Bacci, Elena Sabattini, Carlo Sagramoso, Roberto Piva, Fernando Roncolato, Giorgio Inghirami, Stefano A. Pileri

**Affiliations:** ^1^Hematopathology Section, Department of Hematology and Oncological Sciences “L. and A. Seràgnoli”, S. Orsola-Malpighi Hospital, University of Bologna, 40138 Bologna, Italy; ^2^Molecular Pathology Laboratory, Haematopathology Section, Department of Hematology and Oncological Sciences “L. and A. Seràgnoli”, S. Orsola-Malpighi Hospital, University of Bologna, Pavillon 8, Via Massarenti 9, 40138 Bologna, Italy; ^3^Center for Experimental Research and Medical Studies (CERMS), University of Torino, 10126 Torino, Italy; ^4^Department of Haematology, St. George Hospital, Clinical Services Building, Kogarah NSW 2217, Australia

## Abstract

The authors revise the concept of anaplastic large cell lymphoma (ALCL) in the light of the recently updated WHO classification of Tumors of Hematopoietic and Lymphoid Tissues both on biological and clinical grounds. The main histological findings are illustrated with special reference to the cytological spectrum that is indeed characteristic of the tumor. The phenotype is reported in detail: the expression of the ALK protein as well as the chromosomal abnormalities is discussed with their potential pathogenetic implications. The clinical features of ALCL are presented by underlining the difference in terms of response to therapy and survival between the ALK-positive and ALK-negative forms. Finally, the biological rationale for potential innovative targeted therapies is presented.

## 1. Introduction

Anaplastic large cell lymphoma (ALCL) is a peripheral T-cell-derived malignancy, representing around 2%-3% of all lymphoid neoplasms, according to the World Health Organization (WHO) estimates [1, 2]. Originally described by Stein et al. in 1985 [3], it has undergone a series of revisions, which have led to a more refined and restrictive definition of the process [1, 2, 4, 5]. In particular, two different entities are recognized as systemic forms, the ALK^+^ and ALK^−^ ALCL [1, 2, 6], on genetic and clinical features, the first one being characterized by the deregulated expression of chimeric proteins expressing the intracytoplasmic domain of the anaplastic lymphoma kinase (*ALK*) gene. Noteworthy, in the last edition of the WHO classification, ALK^−^ ALCL was regarded only as a provisional entity [1, 2]. However, emerging evidences suggest the existence of two real tumors [7]. On the other hand, differently from what initially reported by Stein et al. [3], the cutaneous variant was recognized as a different disease [8].

Primary systemic ALCL has a peak incidence in childhood, accounting for approximately 40% of NHL cases diagnosed in pediatric patients [9], whereas it accounts for <5% of NHL in adults [1, 2, 10], and it is seen mostly in males. Patients present with stage III to IV disease, often with multiple extranodal sites of involvement [1, 2, 11, 12].

In this article, the authors, based on their own experience and the most recent literature, review the main pathobiological features of systemic ALCL, by focusing on the molecular abnormalities representing potential targets for innovative therapies. 

## 2. Morphology

According to the WHO classification, ALCL is not sustained by a unique histotype but actually includes five morphologic variants (common, giant cell-rich, lympho-histiocytic, small-cell type, and Hodgkin-like) [1, 2, 13, 14]. Indeed, common ALCL corresponds to the description of the tumor given by Stein and coworkers in 1985 [3].

**Figure 1:**
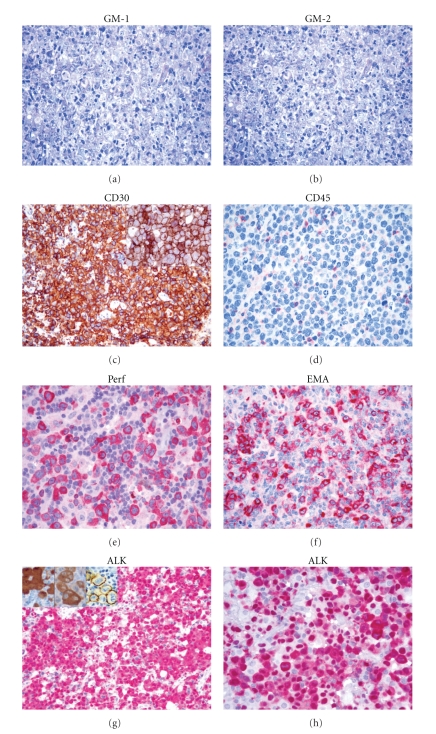
Morphological and immunophenotypical features of ALCL. At morphology (GM), a variable amount of hallmark cells can be identified, the phenotype being typically CD30^+^, and possibly CD45^−^, EMA^+^, and Perforin (Perf)^+^. In ALK^+^ cases, ALK staining more frequently interests both nucleus and cytoplasm, being associated with *NMP1/ALK* translocation. However, different transcripts may determine an isolated cytoplasmic or membranous staining (see insets; refer to Table 1 for details).

All morphological variants are characterized by a variable proportion of large hallmark cells with eccentric horse-shoe or kidney-shaped nuclei, often with eosinophilic region near the nucleus (Figure 1). The giant cell-rich type is characterized by several multinucleated elements, often provided with Reed-Sternberg-like features and prominent intrasinusoidal diffusion [1, 5, 15, 16]. The small and lympho-histiocytic variants display a marked variability of the neoplastic cell size that ranges from small to large. The main difference between the two forms is an aboundant reactive histiocytosis with eccentric nucli in the latter [1, 2, 13, 17–19]. Such a reactive population tends to obscure the neoplastic component and can lead to a misdiagnosis of hyperimmune reaction [1, 2, 13, 17, 18, 20]. Interestingly, transition from one histotype to the other is at times recorded within the same node (mixed variant) or in different nodes taken from the same patient at the time of diagnosis or in relapse: these modifications might correspond to intraclonal modulation or different interaction between the tumor and microenvironment [13, 21]. Further variants have been reported in the literature: signet-ring cell-like, sarcomatoid, epitheliod cell-rich, and eosinophilic-rich [13, 22, 23]. They are much rarer than the previous ones and may indeed represent a diagnostic challenge. Finally, the so-called ALCL of the Hodgkin-like type deserves special attention [24]. It was originally described as a form of the tumor, presenting in young people with a bulky mediastinal mass and consisting of anaplastic cells arranged in nodules surrounded by sclerotic bands, as seen in nodular sclerosing Hodgkin's lymphoma (NSHL) [24]. Following the introduction of the REAL Classification [5], which regarded it as a provisional entity, such diagnosis was by no means also applied to cases of aggressive HL that could not be easily differentiated from ALCL, both on morphologic and on phenotypic grounds [5]. This led to a diffuse skepticism on the existence of such variant: it was considered an undefined group more than an entity. However, *bona fide* examples of ALCL of the Hodgkin's-like type can be encountered. These are characterized by ALK protein-expression, homogeneous CD30-positivity, lack of CD15 and B-cell activator protein (BSAP), possible T-cell antigen expression, and variable positivity for the leukocyte common antigen/CD45 and epithelial membrane antigen (EMA) (see below).

Only basing on morphology and phenotype, the ALK^−^ ALCL is not reproducibly distinguishable from the ALK^+^ variant. The two neoplastic proliferations show, in fact, a similar morphological spectrum, although the small cell variant is not recognized in ALK^−^ ALCL. The neoplastic cells, in ALK^ −^ ALCL tend to be larger and more pleomorphic, with higher nuclear/cytoplasmatic ratio than those seen in classical ALK^+^ cases. This feature may suggest a diagnosis of peripheral T-cell lymphoma not otherwise specified (PTCL/NOS), but in the latter disorder, small-medium-sized lymphocytes are often admixed with a morphological homogeneous neoplastic cell proliferation and sheet-like or sinus pattern of infiltration typical of ALCL is absent. Lastly, ALK^−^ ALCL must be distinguished from primary cutaneous ALCL which can have a similar phenotype and morphology. Thus, the clinical correlation with staging is golden rule in those cases because primary cutaneous ALCL has a much better prognosis than ALK^−^ ALCL. 

## 3. Phenotype

**Figure 2:**
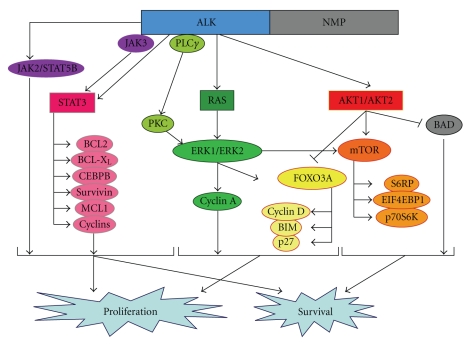
Schematic representation of ALK signalling. In particular, ALK signalling promotes tumor cell proliferation and survival via STAT3, RAS/ERK, and AKT/mTOR pathways. Noteworthy, the blockage of the signalling was demonstrated to have significant antitumor activity in experimental models of ALK^+^ ALCL.

Neoplastic cells of ALCL carry a distinctive phenotypic profile irrespective of the histotype [1, 2, 15, 25]. They regularly express CD30 [1–3, 26] (Figure 1), a glycoprotein of 120 kD carried by lymphoid elements following activation and formed by three distinct domains (intracytoplasmic, transmembranic, and external) [3, 26]. It is encoded by a gene located at 1p36, whose activity is modulated by the number of ATCC-repeats in the 5′ region of the promoter, and represents a member of the receptor superfamily of tumor necrosis factor (TNF) [26]. Notably, CD30 overexpression has been reported to induce constitutive NF-*κ*B activation [27, 28]. As expected, the CD30 ligand (CD30L) belongs to a group of molecules, which show homologies with TNF. The external domain of CD30 is steadily cleaved by a metalloproteinase so that it can be detected and measured in the serum [29]. At immunohistochemical analysis in both in paraffin and frozen sections, the antibodies against CD30 produce different types of positivity: membrane-bound, dot-like in the Golgi area (corresponding to the accumulation of the 90 kD proteic precursor), and diffuse [26]. The first two patterns are exclusive to lymphoid elements with the exception of embryonic carcinoma [26], while the third one can occur in a variety of malignant tumors other than lymphomas, including pancreas carcinoma, naso-pharyngeal undifferentiated carcinoma, mesothelioma, and malignant melanoma [26]. Therefore, the immunophenotypic diagnosis of ALCL should always be based on the application of a panel of antibodies, including reagents anticytokeratins, melanoma-associated antigens, CEA, and PLAP [1, 2]. In 60%–70% of cases, ALCL carries the epithelial membrane antigen (EMA) (Figure 1): this molecule is more easily detected in Bouin-fixed samples [13, 30]. CD3 expression is appreciated in about half cases: it usually occurs at the cytoplasmic level as expected in activated cells [13, 31]. All T-cell associated antigens should be explored, as CD3 negative tumors may carry CD2, CD5, and/or CD7 [13, 15, 31–33]. The expression of CD4 and CD8 is variable [13, 15]. Positivity for TIA-1, granzyme B, and perforin is recorded in about 85% of instances (Figure 1). NK-antigens have at times been detected, in both spontaneous and experimental tumors [34, 35]. About 20% of ALCLs lack CD45 and/or express CD15 [13] (Figure 1). Notably, BSAP (i.e., the PAX5 gene product) is absent [36]: its search represents a very useful tool for the differentiation of ALCL from common HL and DLBCL, which are both BSAP-positive [36]. The recent development of antibodies against the anaplastic large cell lymphoma kinase (ALK) has further refined the immunohistochemical analysis of ALCL [13, 15, 17, 37] (Figure 1). In fact, expression of this molecule does characteristically occur in cases carrying translocations that involve the corresponding gene (see below) [13, 15, 17, 37] (Table 1). In principle, the most common t(2;5) causes strong positivity of the neoplastic cells at the cytoplasmic and nuclear levels, while the other translocations produce accumulation of the protein in the cytoplasm. These different staining patterns correspond to over-expression of the *ALK* gene product: they bear diagnostic relevance, since ALK is detected neither in normal lymphocytes nor in HL [17, 38]. Of note, such kinase has been identified in some lymphoid and nonlymphoid neoplasms that have nothing in common with ALCL [38]. In particular, it can occur—more often at the cytoplasmic level—in B-plasmablastic lymphomas bearing a distinctive phenotype (ALK^+^, CD138^+^, EMA^+^, IgA^+/−^, Bcl-2 protein^+/−^, CD4^−/+^, CD57^−/+^, CD20^−^, CD3^−^, and CD30^−^): like ALCL, these tumors do also carry cytogenetic abnormalities involving the *ALK* gene [38]. Among nonhaematopoietic neoplasms, ALK positivity is found in inflammatory myofibroblastic tumour (IMT) and some neuroblastomas and rhabdomyosarcomas and carcinomas [39, 40]. Interestingly, it has been shown that ALK has immunogenic properties, thus causing the production of antibodies that can be easily detected in the serum and might be relevant to the relatively good prognosis of ALK^+^ ALCL [41]. These immunogenic properties should be taken into account for two additional reasons: (1) they might be employed for vaccination strategies [42] and (2) they might cause extensive tumor destruction, hypocellularity, and oedema of the affected nodes, thus mimicking an inflammatory lesion [43]. Notably, ALK expression is felt to play a relevant role in the process of lymphomagenesis, as suggested by experimental data [44–46]. In this respect, experimental and *in vivo* studies have provided evidence that the chimeric protein NPM/ALK secondary to t(2;5) causes profound deregulation of cell kinetics. In fact, it determines phosphorylation of JAK3 and/or direct STAT3 activation: the later induces expression of TIMP1 and Bcl-X_L_ and Bcl-A2 that in turn with increased activated caspase-3 levels contributes to the activation of the antiapoptotic pathway [46] (Figure 2). In addition, the NPM/ALK chimeric protein facilitates proliferation via activation of a series of factors, including PLC-*γ*, type IA phosphoinositide 3-kinase, Src-kinases, AKT, and FOXO3a [23] (Figure 2). Interestingly, the above mentioned cell-kinetic alterations are not observed to the same extent in many ALK^−^ ALCLs, a fact that further supports the distinction of ALK-positive and negative cases. In line with this, the different expression of the lymphocyte specific protein (LSP1) detected in all ALK^+^ ALCLs and only 25% of the negative ones [47]. Conversely, expression of retinoblastoma protein and survivin can occur in both ALK^+^ and ALK^−^ ALCLs, in any case representing independent unfavorable prognostic indicators [48, 49].

 Finally, the search for Epstein-Barr virus (EBV) is negative in most if not all ALCLs both by *in situ* hybridization (ISH) by and immunohistochemistry: such negativity is regarded as one of the distinguishing features between ALCL and HL in controversial cases [50]. Recently, however, occasional ALK^+^ ALCLs showing EBV integration in their genome have been reported: they occurred in patients with a previous history of solid organ transplant [51]. 

## 4. Molecular Genetics

The t(2;5)(p23;q35) was originally described in examples of malignant histiocytosis (MH) [52]. These actually represented ALCL cases diagnosed according to dated criteria. In fact, it quickly became evident that such translocations characteristically occurred in ALCL [53–56]. At the beginning, the real incidence of the phenomenon was uncertain: in fact, the need for fresh or frozen material for cytogenetic studies or Southern blot analysis prevented its systemic search. In principle, the aberration produced the development of a hybrid gene, formed by the segment of the *ALK* gene encoding for the transmembrane portion of the corresponding kinase and the NH_2_-terminal region of the *NPM1* gene [38, 57]. Under physiologic conditions, the latter encodes for nucleophosmin, that is, a shuttle-protein that undergoes dimerization in the cytoplasm and subsequently moves to the nucleus [58]. Thanks to the production of highly specific (polyclonal and monoclonal) antibodies against the transmembrane domain of the ALK protein, as well as the NH_2_- and COOH-terminal regions of NPM, the identification of the chimeric NPM/ALK protein (also termed p80 because of its molecular weight) became easily feasible in routine (formalin-fixed, paraffin-embedded) samples [17, 37, 59]. The resulting assessment demonstrated that the large majority of cases diagnosed as ALCL according to the REAL/WHO Classification do indeed carry t(2;5) [55, 60]. The staining produced by the antiALK antibodies is typically cytoplasmic and nuclear: this is due to the fact that the NPM/ALK protein forms heterodimers with normal NPM and—like normal NPM homodimers—is shuttled to the nucleus [37, 60, 61] (Figure 1). Unexpectedly, the broad application of the monoclonal antibodies ALK1 and ALK_c_ revealed that about 10% of ALK^+^ ALCLs showed an immunohistochemical reactivity confined to the cytoplasm. This was concomitant with the detection of a series of additional translocations (commonly called variant translocations), all involving the *ALK* gene, but leading to the formation of a chimeric gene with partners other than *NPM1* [38, 62] (Figure 1). The derived hybrid proteins—all leading to deregulated expression of ALK fusion proteins—are more often under oligomerization but have not shuttling properties, thus remaining confined to the cytoplasm [38]. The most relevant of these variant translocations are listed in Table 1.

 Interestingly, one of these variants, t(2;17)(p23;q23), is also observed in IMT as well as in B-plasmablastic lymphoma [63, 64], while t(1;2)(q25;p23), t(2;19)(p23;p13), and t(2;11;2)(p23;p15;q31) are detected more frequently in IMT than in ALCL [63]. Conversely, with the exception of two studies, which results could not be reproduced in other labs, neither ALK protein detection nor t(2;5) or variant translocations have been detected in HL [65].

 Recently, by using a comparative genomic hybridization (CGH) platform, Salaverria et al. [66] identified chromosomal imbalances in 58% of ALK^+^ ALCL and in 65% of ALK^−^ cases, within a cohort of 74 cases (43 ALK^+^ and 31 ALK^−^, resp.). Importantly, ALCL carrying *NPM1-ALK* or other translocations involving *ALK* showed a similar profile of genetic alterations. In particular, recurrent gains of 17p and 17q24 and losses of 4q13-q21 and 11q14 were found in ALK^+^ ALCL. On the other hand, gains of 1q and 6p21 were more frequently observed in ALK^−^ forms [66]. Thus, CGH data confirmed that ALK^+^ and ALK^−^ ALCL are different genetic diseases, though few recurrent chromosomal imbalances were found in both types of tumors (gains of 7 and 6q and 13q losses), confirming that all ALCLs probably share common pathogenetic events (see below) [67, 68]. 

### 4.1. Gene Expression Profiling

Regarding gene expression profiling (GEP), Thompson et al. [67] initially demonstrated the ability of GEP to correctly distinguish between ALK^+^ and ALK^−^ ALCL based on the analysis of their transcriptome. Importantly, in this study it was also suggested that some pathogenetic mechanisms might be shared by these two entities, based on the common expression of certain genes in both ALK^+^ and ALK^−^ cases. 

 Subsequently, the study by Lamant et al. [69] confirmed that the different morphological variants of ALCL (common type, small cell, and “mixed” variants) could be distinguished based on the expression of specific genes. Moreover, it was shown that ALK^+^ and ALK^−^ ALCL have different molecular signatures. Specifically, within the molecular signature of ALK^+^ ALCL* BCL6, CEBPB, SERPINA1, *and* PTPN12* were found to be overexpressed, some of these data being further validated by immunohistochemistry on tissue microarrays. Conversely, ALK^−^ ALCLs were found to overexpress *CCR7, CNTFR, IL22,* and *IL21*. Overall, this study strongly supported the concept that ALK^+^ and ALK^−^ ALCL are different entities but did not provide novel information as far as the molecular pathogenesis of ALK^−^ ALCL was concerned.

 Our group then included some ALCL in a GEP study on PTCLs [68]. Interestingly, we found that ALCL can be roughly distinguished from other PTCLs irrespectively of the ALK status, confirming the idea of common pathogenetic events. Finally, additional important information has been then offered by Piva et al. [70]. In this study, we have mainly focused on the molecular pathogenesis of ALCL but also established the relationship between ALCL and PTCL/NOS. In particular, we showed that ALCLs are molecularly distinct from PTCL/NOS. Significantly, a predictive analysis allowed identification of 34 probe sets capable of distinguishing ALCL from other T-NHL, suggesting that ALCLs share important biopathological features. Furthermore, it was possible to clearly differentiate ALK^+^ and ALK^−^ cases according to their GEPs, based on the expression of selected genes, including GAS1, an ALK dependent molecule [7]. Finally, we demonstrated the strong biological relevance of the ALK/STAT3 signaling in characterizing the global molecular profile of ALK^+^ ALCL [70].

More recently, Eckerle et al. [33] studied isolated cells from ALCL cases. Interestingly, the analysis supported the derivation of ALCL from activated T cells, though it was not possible to identify a specific counterpart [33]. Surprisingly, the authors found that only few genes were differentially expressed between systemic and cutaneous ALCL despite their different clinical behavior, and between ALK^−^ ALCL and classical Hodgkin lymphoma, despite their different cellular origin [33]. 

### 4.2. Besides the NPM-ALK Chimera

Besides the NPM-ALK chimera several other fusions of ALK have been described in lymphoid neoplasms and more recently in epithelial cancers. Among them, TFG gene (TFG-ALKS (short), TFG-ALKL (long), and TFG-ALKXL (extra long)) have been described [38]. In these translocations, different length fragments of the 5^'^ TFG region, all of them containing the TFG coiled-coil oligomerization domain, are fused with the intracytoplasmic domain of ALK, which drives the constitutive ALK kinase activation. Since the TFG-ALK fusion proteins lack nuclear localization, signals are mainly confined to the cytoplasmic compartment. Analogously, the translocation t(1;2)(p23;q21) leads to translation of an another cytoplasmic ALK fusion protein in which the NH-2 terminus region of TPM3 is linked to the intracytoplasmic domain of ALK. ALK fusions also occur with TPM4, a homolog of TPM3, and both TPM3- and TPM4-ALK proteins lead to the constitutive autophosphorylation of ALK as a result of homodimerization through the TPM coiled-coil domains [38].

ATIC, CLTCL and MSN-ALK are also nonnuclear ALK fusion chimeras. In ATIC-ALK, the 5-aminoimidazole-4-carboxamide ribonucleotide formyltransferase/IMP cyclohydrolase (ATIC) gene, located within chromosome 2, which catalyzes steps of de novo purine nucleotide biosynthesis, is fused to ALK as a result of cryptic inversion inv(2) 9p23q35 [38]. ALK can be fused to the CLTCL (clathrin heavy polypeptide-like) gene (17q23) and CLTCL-ALK proteins display a typical cytoplasmic dot-like expression pattern reflecting an ALK-positive polyhedral coat on the surface of vesciles [38]. Finally two other translocations, the t(2;17)(p23;q25) and the t(2;22)(p23;q11.2), lead to the translation of AL017- and MYH9-ALK chimera which localized within the cytoplasms. A different cell localization is documented for the moesin- (MSN-) ALK fusion protein, whose restricted surface membrane distribution is probably attributable to association with wild-type MSN and other membrane proteins. MSN-ALK does not contain an oligomerization domain in its N-terminal domain. 

Despite different structural and cellular localization all ALK fusion proteins have very similar transforming properties *in vitro* and no clear clinical differences have been so far described. 

Together, these findings tend to suggest that the transforming properties of ALK fusion could be due to their cytoplamic localization and/or to activation of multiple downstream molecules primarily localized within the cytoplasmic compartment. 

## 5. Clinical Behavior

ALCL is an aggressive lymphoma which frequently presents in advanced clinical stage (III-IV) with systemic symptoms, and extranodal involvement, as other PTCLs do [15, 60]. Bone marrow involvement is detected in up to 30% of cases, being a relevant prognostic feature [71–73]. 

 Importantly, ALCLs display quite different clinical features depending on the expression of the ALK protein [6, 15, 60, 74–76]. In particular, ALK^+^ tumors most frequently occur among patients in the first or second decade of life, while ALK^−^ ones are usually recorded among people aged 50–70 [6, 15, 60]. Moreover, advanced-stage disease and B symptoms are slightly more common in ALK^+^ ALCL [6, 15]. Most importantly, several studies have shown that ALK^+^ has a significantly better outcome than ALK^−^ ALCL. In particular, up to 90% of ALK^+^ ALCLs achieve complete remission (CR) by adopting standard antracyclin-containing regimens, and 70%–80% of patients were actually cured [6, 15, 60, 74–76]. By contrast, only around 30%–50% of ALK^−^ cases obtain stable CR by the same therapies [6, 15, 60], suggesting that more aggressive strategies including autologous or allogenic bone-marrow/stem cell transplantation may be necessary [77]. Interestingly, leukemic spread seems to represent a major exception to the favorable prognosis of ALK^+^ ALCL [6, 15, 60, 72].

 Noteworthy, the International T-cell lymphoma project has recently reported that ALK^+^ and ALK^−^ ALCL seem to have a similar prognosis (in terms of both FFS and OS), when patients are stratified according to the clinical parameters (i.e., age and/or stage). Grippingly, this would suggest a prominent role for clinical factors in determining patients outcome, rather than for biological components [6]. 

 Notably, in the past years, it was suggested that the distinction between ALK^−^ ALCL and peripheral T-cell lymphoma, not otherwise specified (PTCL/NOS), was of limited clinical relevance, only age and the International Prognostic Index (IPI) being of prognostic relevance in these tumors [78]. Nevertheless, it was recently shown that clinical differences do exist between the two entities. In particular, in a large international study, a greater proportion of patients with poor performance status and B symptoms, but a lower frequency of bone marrow invasion, splenic involvement, and thrombocytopenia, was observed with ALK^−^ ALCL as compared to PTCL/NOS [6]. Indeed, in this study, ALK^−^ ALCL showed an overall outcome significantly better than PTCL/NOS [6]. 

## 6. Targeted Therapy

Despite the recent significant progresses in understanding the molecular pathogenesis of ALCL, the therapeutic approaches are still based on doxorubicin-containing combination chemotherapy [6]. However, the fact that NPM-ALK plays a central role in the development and progression of ALK^+^ ALCL tumors makes it an ideal candidate therapeutic target in this disease. Notably, the wt ALK protein is not widely expressed in adult tissues, being abundant only in a few neuronal cells. Therefore, few toxic effects might be expected from treatment aimed at blocking ALK function. Remarkably, the administration of selective inhibitors induced tumor growth arrest and possibly its regression *in vivo* [79, 80]. More recently, ALK was shown to be an ideal candidate target for antitumor vaccination. Indeed, in ALCL xenograft models, such strategy completely prevented tumor growth and increased the percentages of cured mice when combined with chemotherapy [42].

 Additionally, the JAK/STAT and the PI3K/AKT have been recently proposed as potential targets in ALK^+^ lymphomas [46, 81, 82]. Similarly, the blockage of their activation by interfering with the upstream cytokines and growth factors has been shown to be potentially effective [83]. Importantly, as tyrosine-kinase deregulation has been documented in several other T-NHLs [68, 84–86], it is conceivable that ALK^−^ ALCL might present similar phenomenon as well, further studies being indeed warranted.

 Finally, immunotherapy strategies could represent another possible therapeutic approaches. In particular, *in vitro* and *in vivo* studies showed that antiCD30 antibodies induce apoptotic cell death and tumor regression in CD30^+^ lymphomas, including ALCL [87–91]. In addition, other studies have suggested that CD26 might represent a promising immunotherapeutic target [92]. 

## 7. Conclusion

In conclusion, based on the most recent findings, the WHO classification currently considers two ALCL types, ALK^+^ and ALK^−^. In fact, though the latter is still quoted as a provisional entity, increasing evidence, both biological and clinical, suggests the real existence of two distinct, though similar, tumors. ALK represents an ideal therapeutic target for innovative strategies, including small inhibiting molecules and even vaccination. On the other hand, ALK^−^ cases may benefit, in the future, from monoclonal antibodies (i.e., antiCD30), tyrosine-kinase inhibitors, or other signal transduction inhibitors. 

## Figures and Tables

**Table 1:** Main translocation involving ALK recorded in anaplastic large cell lymphoma.

Chromosomal translocation	Partner gene	Fusion gene	Frequency (5)	ALK IHC detection
t(2;5)(p23;q35)	*NPM1*	*NPM1/ALK*	75–80	Cytoplasmic/nuclear
t(1;2)(q25;p23)	*TMP3*	*TMP3/ALK*	12–18	Cytoplasmic
inv(2)	*ATIC*	*ATIC/ALK*	2	Cytoplasmic
t(2;3)(p23;q21)	*TGF*	*TGF/ALK*	2	Cytoplasmic
t(2;17)(p23;q23)	*CLTL*	*CLTL/ALK*	2	Cytoplasmic
t(2;17)(p23;q25)	*ALO17*	*ALO17/ALK*	<1	Cytoplasmic
t(2;19)(p23;p13)	*TPM4*	*TPM4/ALK*	<1	Cytoplasmic
t(2;22)(p23;q11.2)	*MYH9*	*MYH9/ALK*	<1	Cytoplasmic
t(2;X)(p23;q11-12)	*MSN*	*MSN/ALK*	<1	Membranous

*NPM1*: nucleophosmin; *ALK*: anaplastic lymphoma kinase; *TPM3*: tropomyosin 3; *ATIC*: 5-aminoimidazole-4-carboxamide ribonucleotide formyltransferase/IMP cyclohydrolase; *TGF*: TRK-fused gene; *CLTL*: Clathrin heavy chainlike 1; *ALO17*: ALK lymphoma oligomerization partner on chromosome 17; *TPM4*: tropomyosin 4; *MYH9*: Nonmuscle myosin heavy chain; *MSN*: moesin; IHC: immunohistochemistry.
