# Correction to “Pathobiology of Anaplastic Large Cell Lymphoma”

**DOI:** 10.1155/ah/9754703

**Published:** 2026-07-28

P. P. Piccaluga, A. Gazzola, C. Mannu, et al., “Pathobiology of Anaplastic Large Cell Lymphoma,” *Advances in Hematology*, vol. 2010 (2010), https://doi.org/10.1155/2010/345053.

In the article titled “Pathobiology of Anaplastic Large Cell Lymphoma,” there was an error in Figure 1. Specifically, the image in panel (a) contained overlapping features with panel (b). The error was introduced by the authors during figure assembly and Figure 1 should be corrected as follows:

**FIGURE 1:**
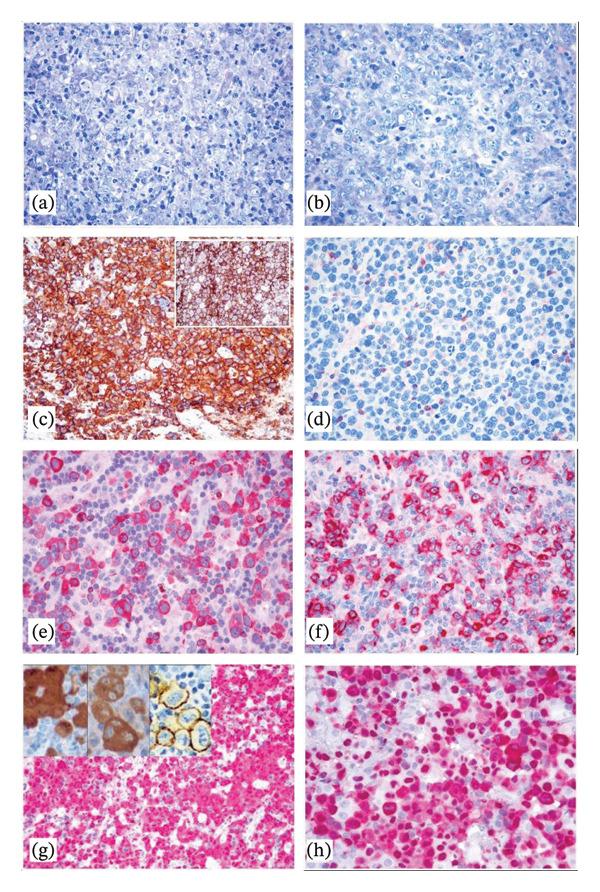
Morphological and immunophenotypical features of ALCL. At morphology (GM), a variable amount of hallmark cells can be identified, the phenotype being typically CD30+, and possibly CD45−, EMA+, and Perforin (Perf)+. GM1 (a) and GM2 (b) show the morphologic spectrum of ALK + ALCL, GM1 showing an amount of small neoplastic cells besides typical hallmark elements and GM2 essentially consisting of large anaplastic cells. In ALK + cases, ALK staining more frequently interests both nucleus and cytoplasm, being associated with NMP1/ALK translocation. However, different transcripts may determine an isolated cytoplasmic or membranous staining (see insets; refer to Table 1 for details)].

The authors also wish to clarify that panels (a) and (b) represent the tissue of the same patient/sample and that the images are intended for illustrative purposes. The authors provided additional information to clarify the differences between these panels which can be seen in the revised legend for Figure 1.

We apologize for these errors.

